# Mapping the optimal forest road network based on the multicriteria evaluation technique: the case study of Mediterranean Island of Thassos in Greece

**DOI:** 10.1007/s10661-015-4876-9

**Published:** 2015-10-13

**Authors:** Stergios Tampekis, Stavros Sakellariou, Fani Samara, Athanassios Sfougaris, Dirk Jaeger, Olga Christopoulou

**Affiliations:** Department of Planning and Regional Development, University of Thessaly, Pedion Areos, 38334 Volos, Greece; Department of Agriculture Crop Production and Rural Environment, University of Thessaly, Fytokou Street, N. Ionia, 38446 Volos, Greece; Institute of Forest Sciences, Chair of Forest Operations, Faculty of Environment and Natural Resources, University of Freiburg, Verfahrenstechnik Werthmannstraße 6, 79085 Freiburg i.Br, Germany

**Keywords:** Forest road network, Spatial layout, Environmental impact assessment, Natural resources, Multicriteria evaluation, GIS

## Abstract

The sustainable management of forest resources can only be achieved through a well-organized road network designed with the optimal spatial planning and the minimum environmental impacts. This paper describes the spatial layout mapping for the optimal forest road network and the environmental impacts evaluation that are caused to the natural environment based on the multicriteria evaluation (MCE) technique at the Mediterranean island of Thassos in Greece. Data analysis and its presentation are achieved through a spatial decision support system using the MCE method with the contribution of geographic information systems (GIS). With the use of the MCE technique, we evaluated the human impact intensity to the forest ecosystem as well as the ecosystem’s absorption from the impacts that are caused from the forest roads’ construction. For the human impact intensity evaluation, the criteria that were used are as follows: the forest’s protection percentage, the forest road density, the applied skidding means (with either the use of tractors or the cable logging systems in timber skidding), the timber skidding direction, the visitors’ number and truck load, the distance between forest roads and streams, the distance between forest roads and the forest boundaries, and the probability that the forest roads are located on sights with unstable soils. In addition, for the ecosystem’s absorption evaluation, we used forestry, topographical, and social criteria. The recommended MCE technique which is described in this study provides a powerful, useful, and easy-to-use implement in order to combine the sustainable utilization of natural resources and the environmental protection in Mediterranean ecosystems.

## Introduction

Because of the environmental impacts, forest road network planning needs to address from ecological aspects. In recent years, the public awareness about the impacts of forest roads on the environment has been increased (Akay et al. 2012; Cole and Landres 1996; Gumus et al. 2008). In order to achieve the sustainable management of the forests, environmental assessments have to be included on scientific and technical principles (Makhdoum 2008). A multifunctional road network planning is prerequisite for the sustainable management and use of forest resources (wood production, ecotourism, water supply, or soil conservation) (Abdi et al. 2009; Demir 2007; Lugoa and Gucinski 2000; Gaodi et al. 2010). Forest roads may cause sediment yield and pollution of off-site water (Arnaez et al. 2004; Forsyth et al. 2006; Fu et al. 2010; Jordán-López et al. 2009; Ramos-Scharro’n and MacDonald 2007) and also ecological fragmentation and disturbance in forest landscapes (Delgado et al. 2007; Forman et al. 1997). Additionally, forest roads can cause losses in habitats and changes in the landscapes (Forman and Alexander 1998; Geneletti 2003). Finally, forest road construction and maintenance have the potential to be the most costly and destructive activities in forest operations (Akay 2006; Larsen and Parks 1997; Najafi et al. 2008) especially if the fundamental design standards are not implemented. The forest road network planning for multiple objectives depends on three objective function (life cycle costs, adverse ecological effects, and landing attractiveness) (Stückelberger et al. 2006) needs, therefore, a new method for the forest road planning that includes financial, ecological, and social parameters needs to be developed (Akay et al. 2008; Aruga et al. 2005; Dutton et al. 2005; Heinimann 1996). Consequently, a functional approach of forest road planning and for optimization of relative parameters (economic and environmental) is necessary (Eker et. al. 2010; Stückelberger 2006). During the previous decades, the basic principles of forest road planning were based on the lowest logging and skidding cost (Aruga 2005; Chung and Sessions 2001; Liu and Sessions 1993; Murray 1998). Some researchers focused on the forest road network planning took under consideration the ecological consequences as well as with the use of multicriteria evaluation method for road planning, such as timber volume, slope, ground condition, distance from existing forest roads, soil type, geology, hydrographic, aspect, elevation, and tree type (Çalışkan 2013; Hosseini and Solaymani 2006; Jusoff 2008; Mohammadi Samani et al. 2010; Sadek et al. 1999). An expert-based approach to the forest road network planning can be achieved by combining the spatial multicriteria evaluation and the Delphi method. This methodology is useful in forest road planning because it takes under consideration environmental and cost parameters (Hayati et al. 2012). Additionally, the application of sensitivity analysis is also a significant method in forest road network planning and environmental impact assessment (Hayati et al. 2013). Likewise, the geographic information systems (GIS)-based multiple-criteria decision analysis (MCDA) (Greene et al. 2011) and the GIS-multicriteria evaluation (MCE) based model for forest road planning (Abdi et al. 2009) are also methods in forest road planning.

The MCE method is a fundamental approach for screening and selecting spatially differentiated decision variants (Beinat and Nijkamp 1998; Jankowski et al. 2008; Voogd 1983). The MCE technique allows us to combine a set of criteria to achieve a decision according to a specific objective (Eastman et al. 1995). The advantage of MCE is that it provides a flexible way of dealing with qualitative multidimensional environmental effects of decisions (Munda et al. 1995). In the last 15 years, much work has been directed toward integrating GIS and MCE methods in the context of spatial decision support systems for planning, retail and service locations, land-based project selection, and environmental management (Eastman et al. 1993; Gomes and Lins 2002; Jankowski 1995; Joerin et al. 2001; Laaribi et al. 1996; Malczewski 1999; Marinoni 2005; Pereira and Duckstein 1993). Nevertheless, over the last 20 years, spatial MCE has come to be recognized as an essential component of spatial decision support system (SDSS) (Malczewski 2006). Integrating GIS-based data processing and analysis techniques and multicriteria decision analysis, we move into the concept of multicriteria spatial decision support system (MC-SDSS) (Malczewski 1999).

In order to evaluate the spatial layout for the optimal forest road network and the environmental impacts that are caused to the natural environment, the intensity and the absorption criteria method could be used (Doukas 2004; Gianoulas 2001; Heinimann 1994).

## Materials and methods

**Fig. 1:**
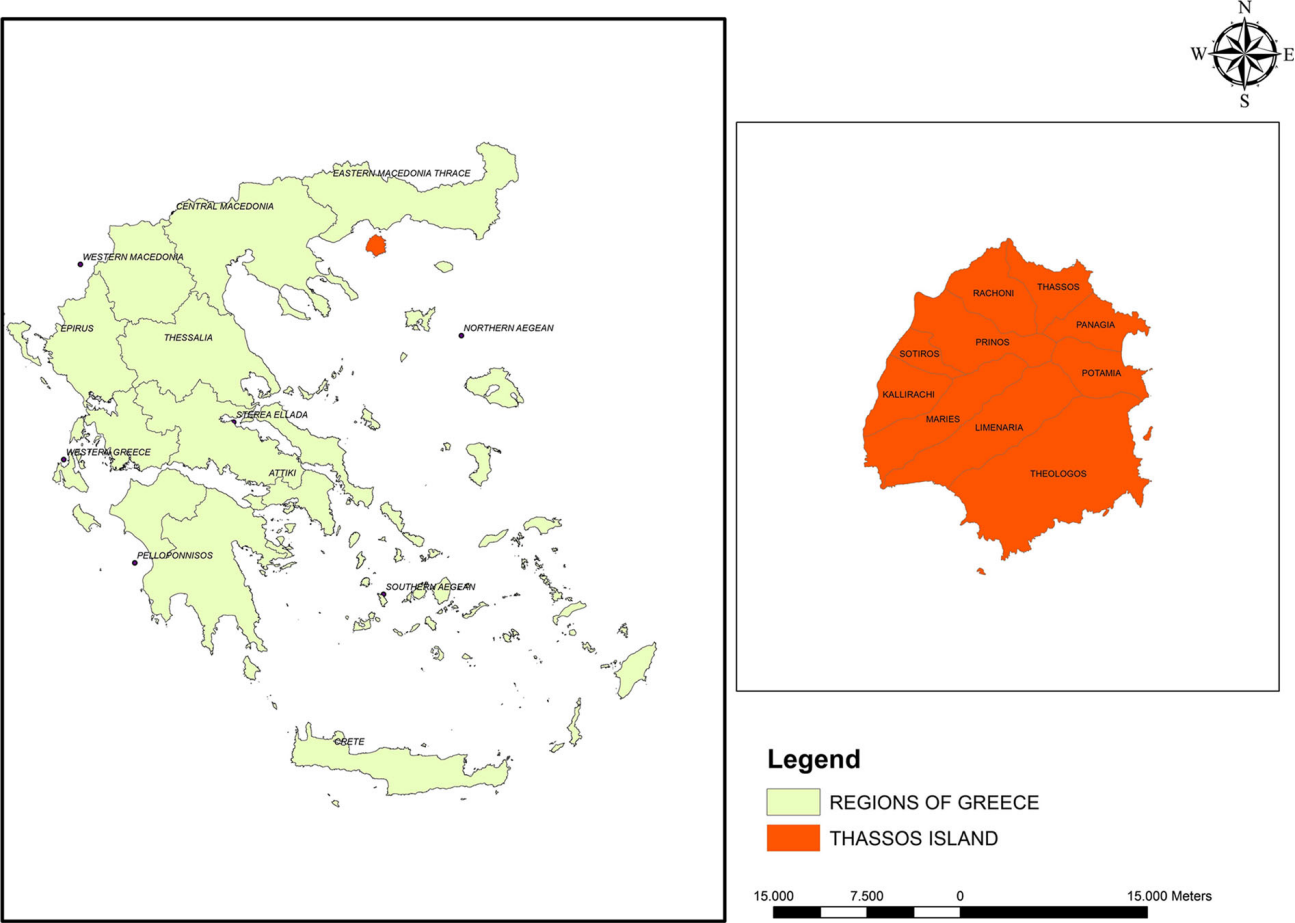
Location of the study area, Thassos Island

At the orientation map (Fig. 1), the study area is presented. As study area, we chose the Greek Island of Thassos (Fig. 1). Specifically, the study area is located at 40.5495 and 40.8351 northern latitude and between 24.4808 until 24.797 western longitude. The study area is about 38,683 ha. The forest of the Thassos Island is non-productive because of forest fires.

For the needs of the research, we used the following: the ArcGIS software, digital orthophotomaps of the area, and respective digital elevation models (DEM). In addition, land use and forest road networks were digitized. We also used the forest management plan for the Island of Thassos for the years 2011–2020.

The MCE technique which is described is a recommended method that includes the evaluation of the intensity criteria (the forest’s protection percentage, the forest road density, the applied skidding means, skidding direction, visitor’ number and truck load, forest roads’ location) and absorption criteria (forestry, topographical, and society criteria) in order to achieve the spatial layout for the optimal forest road network. The evaluation criteria of intensity refer to the environmental impacts that are caused by the forest roads to the forest ecosystem. The evaluation criteria of forest ecosystem absorption refer to the ability of the environment to absorb the impacts that are caused by forest roads. This method refers to non-productive forests, and we take under consideration the values of the national forestry characteristics.

For the forest protection percentage evaluation, we took under consideration that the forest roads can be used by the firefighting vehicles for the forest protection due to their direct access to the wildfires. The firefighting vehicles of the Greek Fire Service uses are small pickup trucks (4 × 4) equipped with water tanks, piping, and pumps that have the ability to eject water with pressure at 300 m uphill and 500 m downhill from forest roads. Thus, the forest opening-up percentage can be used as the forest protection percentage as well, due to fact that the firefighting vehicles can be utilized for the wildfires’ prevention and suppression.

We agreed on the optimal ecosystem forest protection status to be the 100 %.

For the assessment of the intensity of the environmental impacts, we used the criteria below (Doukas 2004; Gianoulas 2001). Each criterion is rated with a weighting factor (based on experts’ agreements) that represents the intensity and the absorption value.

### Intensity criteria for non-productive forests

The intensity criteria that were used are as follows:

- *Road density and forest protection percentage*. The percentage of the excess or the reduction of the values *D* = 12.5–15 m/ha, forest roads spacing *S* = 667–800 m, and the forest protection percentage, which is <85 %, is rated totally as the reduction of the optimum 100. Weighting factor, 3
- *Applied skidding means*. The percentage of the trees’ skidding that are not extracted with the use of cable logging systems or with draught animals or with the combination of them is rated as the reduction of the optimum 100. Weighting factor, 2
- *Skidding direction (draught animals, cable logging systems)*. The skidding direction percentage which is not achieved in diagonal or in parallel layout, comparing to theoretical skidding distance, is rated as the reduction of the optimum 100. Weighting factor, 1
- *Visitor’ number and truck load.*
•The excess percentage of the visitors’ number, in comparison to the reception capacity of the space (based on the fact that the number of Thassos habitats can visit the forest without causing impacts), is rated as the reduction of the optimum 100. Weighting factor, 2.
•The excess percentage of trucks overloading, which is larger than the permitted by the national regulations, is rated as the reduction of the optimum 100. Weighting factor, 2
- *Forest roads’ location.*
  - The forest roads’ distance from the main streams should be enough not to affect the forest ecosystem. The percentage of forest roads that pass through the valley and the distance from the margins of the main streams which is less than 20 m are rated as the percentage reduction of the optimum 100. Weighting factor, 3
  - The percentage of the roads that pass through in less than 10 m outside the forests’ boundaries or 20 m within the forests’ boundaries is rated as the percentage reduction of the optimum 100. Weighting factor, 3
  - Forest roads shall not be located on sites with unstable soils where they may be slipping, be sliding, or have failures in the construction of embankments. The layout design rate of forest road, passing through unstable soil, large exposure streams, is rated as a percentage reduction of the optimum 100. Weighting factor, 3

The weighted average of the environmental impacts’ intensity evaluation (∑*Ι*) is equal to the sum of the products ∑(*Ι* × *W*_*Ι*_) divided by the sum of the weighting factors (∑*W*_*Ι*_).

1 $$ {\displaystyle \sum I}={\displaystyle \sum \left(I\times {W}_I\right)}\;/\;{\displaystyle \sum {W}_I} $$

where

*Ι* = the criterion value assessment (%) that evaluates the impact intensity which is not negative,

*W*_*Ι*_ = the weighting factor of each intensity criterion

∑*W*_*Ι*_ = the sum of the weighting values of each intensity criterion

### Absorption criteria in non-productive forests

The ability of the forest ecosystem absorption of the forest roads’ impacts was also studied. Specifically, the term absorption is defined by whether the impact effect will be absorbed from the forest ecosystem as time passes, as well as the number of impact receivers. The evaluation criteria of absorption that were studied and the respective weighting factors are as follows:

#### Forestry criteria. Weighting factor, 3

- Land uses: forest, 100 %; forest area, 25–50 %; and barren, 15 % (forest meaning is different from forest area in Greece by national regulations; forest area refers to the areas that have not only trees but shrubs and bushes).
- Forest species: mixed, 100 %; broad-leaved, 75 %; and coniferous, 65 %.
- Forest management form: high forest, 100 %; coppice forest, 50 %; and composite forest, 75–100 %.
- Forest age: group-selective forest, 100 %; gardening forest, 75 %; and even aged forest, 50 %.
- Tree height: high >20 m, 100 %; medium 10–20 m, 75 %; and low <10 m, 25–50 %.
- Plant index groups: I–II, 100 %; III–IV, 50 %; and V–VI, 25 % (the distinction of plant sociological units was performed with the assistance of plant index groups as they are described by Schlenker 1950 and Ellenberg 1956, 1963, 1979).
- Forest productivity (annual growth): high >3 m^3^/year × ha, 100 %; medium 1–3 m^3^/year × ha, 50 %; and low <1 m^3^/year × ha, 25 %.

#### Topographical criteria. Weighting factor, 2

- Slopes: low <8 %, 100 %; medium 8–20 %, 50 %; and high >20 %, 5–25 %.
- Aspects:
  −Elevations <1000 m; northern, 100 %; eastern, 75 %; western, 75 %; and southern, 50 %,
  −Elevations ≥1000 m; northern, 70 %; eastern, 100 %; western, 100 %; and southern, 70 %,
- Terrain relief: mild, 100 %; various, 50 %; and intense (divided into many parts), 15 %.

#### Social criteria (number of receivers). Weighting factor, 1

- The tourist resort.
- The national road network.
- The railway network.
- The archaeological area.
- The neighboring city.
- The neighboring village.
- The European pathway.
- The natural or artificial lake or river.

The rating of the criteria above depends on the number of people that accept the effect and is rated 25 % if the receivers are many, 50 % if the receivers are a few, and 100 % if there are not any.

The weighted average of the environmental impact absorption evaluation (∑*Α*) is equal to the sum of the products ∑(*Α* × *W*_*Α*_)) divided by the sum of the weighting factors (∑*W*_*Α*_).

2 $$ {\displaystyle \sum A={\displaystyle \sum \left(A\times {W}_A\right)\;/\;{\displaystyle \sum {W}_A}}} $$

where

*Α* = the criterion value assessment (%) that evaluates the absorption,

*W*_*Α*_ = the weighting factor of each absorption criterion,

∑*W*_*Α*_ = the sum of the weighting values of each absorption criterion

## Results and discussion

### The rates of the intensity criteria

The rates of each intensity criterion of impacts that are caused from forest roads’ construction to the natural environment are as follows:

### Road density and forest opening up percentage

**Fig. 2:**
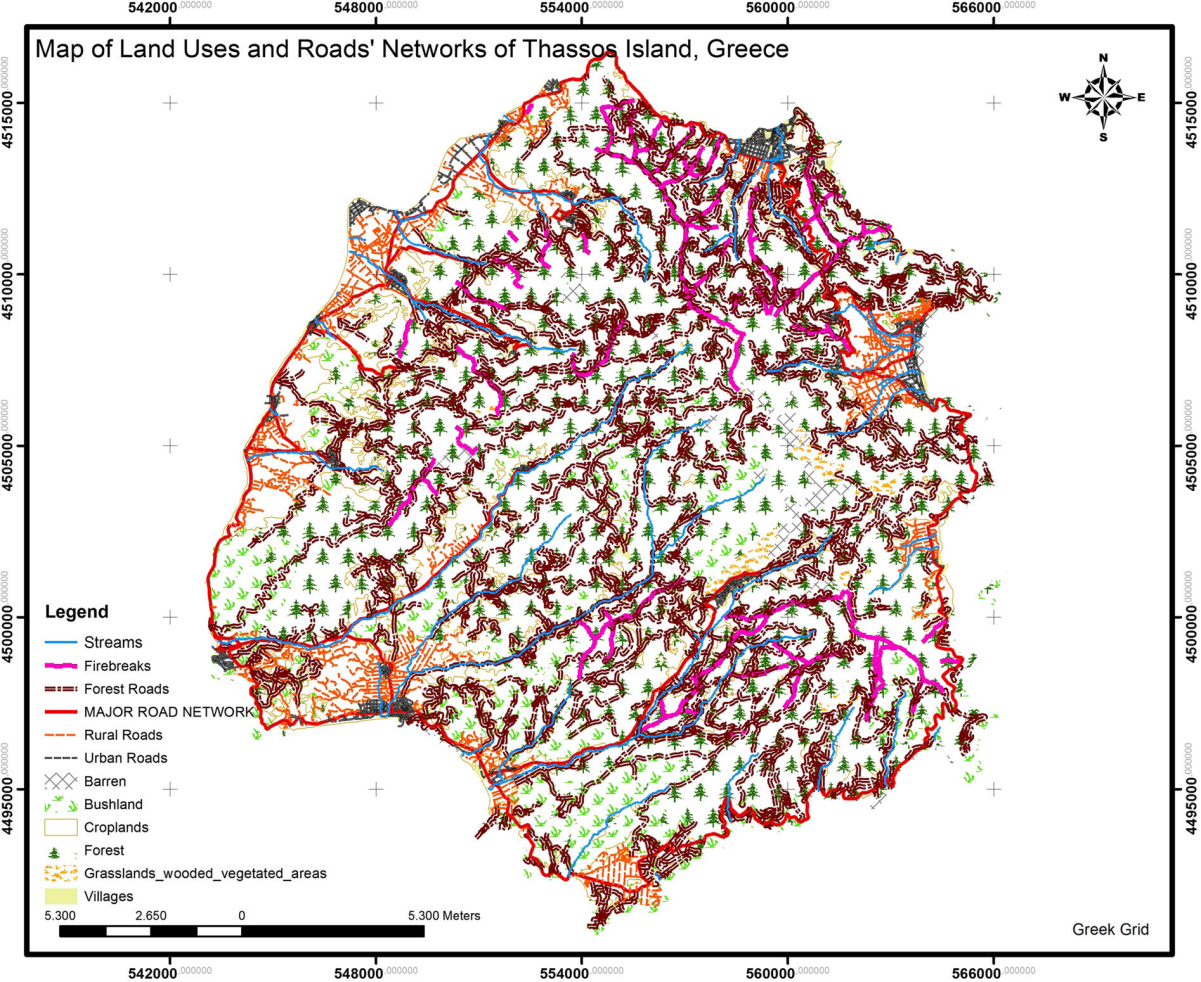
Land uses and roads’ networks of the study area, Thassos Island

**Fig. 3:**
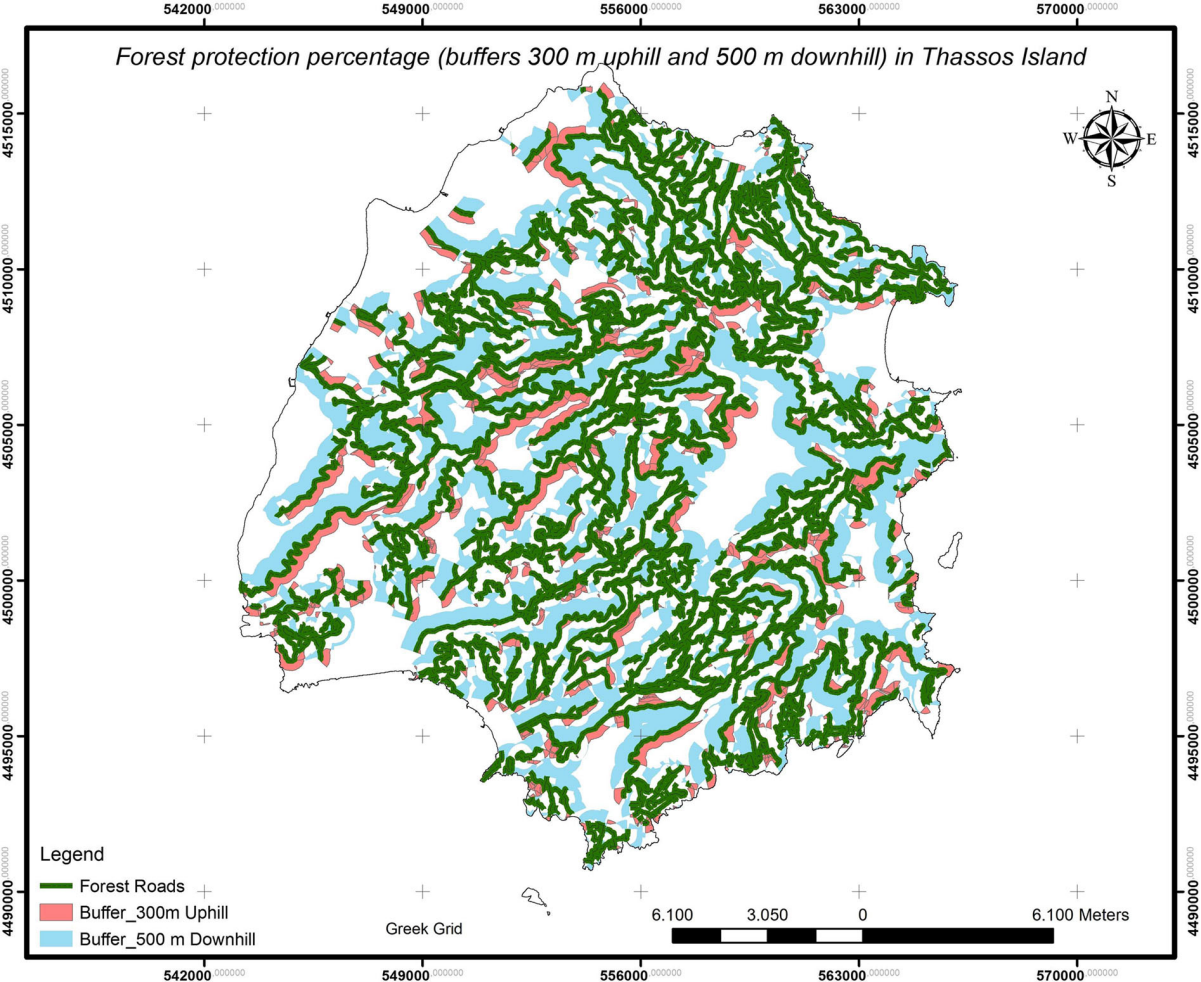
Percentage of forest protection in Thassos Island

**Fig. 4:**
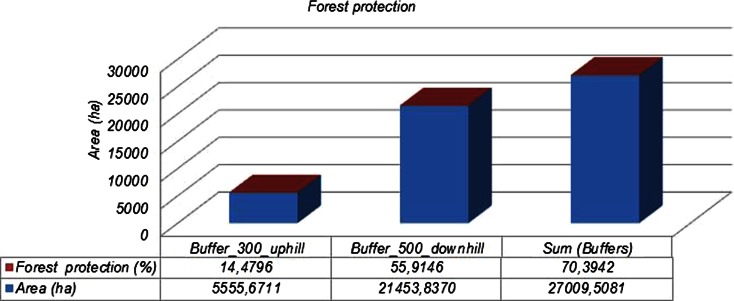
Forest protection percentage

The assessments of (i) the forest road density for the island of Thassos is *D*_ex_ = *L*/*F* = 36.5955 m/ha, where *L* = main forests roads’ length (m) and *F* = forests’ area (ha) (Fig. 2) and (ii) the percentage of forest protection is *E* = 70.39 % (Figs. 3 and 4). With the ArcGIS software, we create buffers (300 m uphill and 500 m downhill from forest roads), and as a result, we have the forest protection map (Fig. 3) and the geodatabase.

So, the excess from the road density values (*D* = 12.5–15 m/ha) is 36.5955 − 12.5 = 24.0955 m/ha and 36.5955 − 15 = 21.5955 m/ha.

The excess percentage is 100 ∗ 24.0955 / 36.5955 = 65.84 % and 100 ∗ 21.5955 / 36.5955 = 59.01 %.

Their average is (65.84 + 59.01) / 2 = 62.425 %.

The reduction percentage from the forest protection percentage, which is smaller than 85 %, is 85–70.39 = 14.61 %.

Finally, the sum is 62.425 + 14.61 = 77.035 %. This percentage is totally rated as the reduction percentage from the optimum 100.

Concluding, the value of the criterion is evaluated 100–77.035 = 22.965 %. Weighting factor, 3

**Fig 5:**
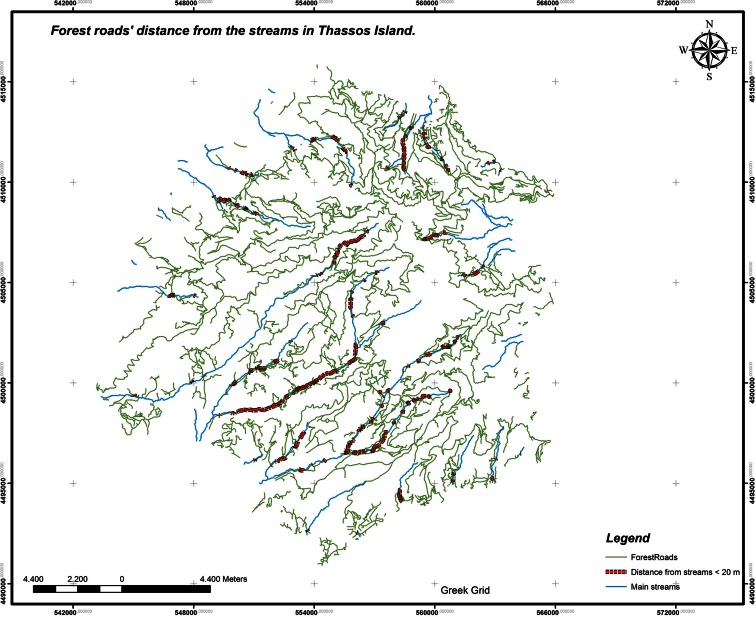
Forest roads’ distance from the streams in Thassos Island

- *Applied skidding means.*
- The forests in the study area have not been productive during the last 25 years and do not produce timber for any use because of the fires that had broken out in the decade of 1980. Thus, the skidding means are not used for timber skidding. This criterion is therefore not rated. Weighting factor, −
- *Skidding direction (draught animals, cable logging systems).*
- This criterion is not rated because of the fact that in the study area, wood skidding has not been carried out due to the forest protection and the unproductive management. Weighting factor, −
- *Visitor’ number and truck load.*
  - In the study area, there is not any excess of the visitors’ number, in comparison to the reception capacity (based on the fact that the number of Thassos habitats can visit the forest without causing impacts) of the space. So, the excess percentage is 0 %. The value of the criterion is evaluated as 100 − 0 = 100 %. Weighting factor, 2.
  - In the study area, there is not any truck presence larger than the permitted by the national regulations, due to the fact that the forest in the area has not been productive during the last 25 years. Thus, the percentage of the truck overloading is 0 %. The value of the criterion is evaluated as 100 − 0 = 100 %. Weighting factor, 2
- *Forest roads’ location.*
•For the evaluation of this criterion, ModelBuilder tool of ArcGIS has been used. Finally, the percentage of forest roads (Fig. 5) that pass through the valley and the distance from the margins of the main streams which is less than 20 m are given in Table 1.

**Table 1:** Intensity evaluation rating due to the forest roads’ distance from the streams

Distance from streams (m)	Forest roads’ length (m)	Road percentage (%)
<20	30,708.3571	3.0587
≥20	973,244.0414	96.9413
Total	1,003,952.3985	

The criterion rate for the forest roads’ distance from the streams is evaluated 100 − 3.0587 = 96.9413. Weighting factor, 3

**Fig 6:**
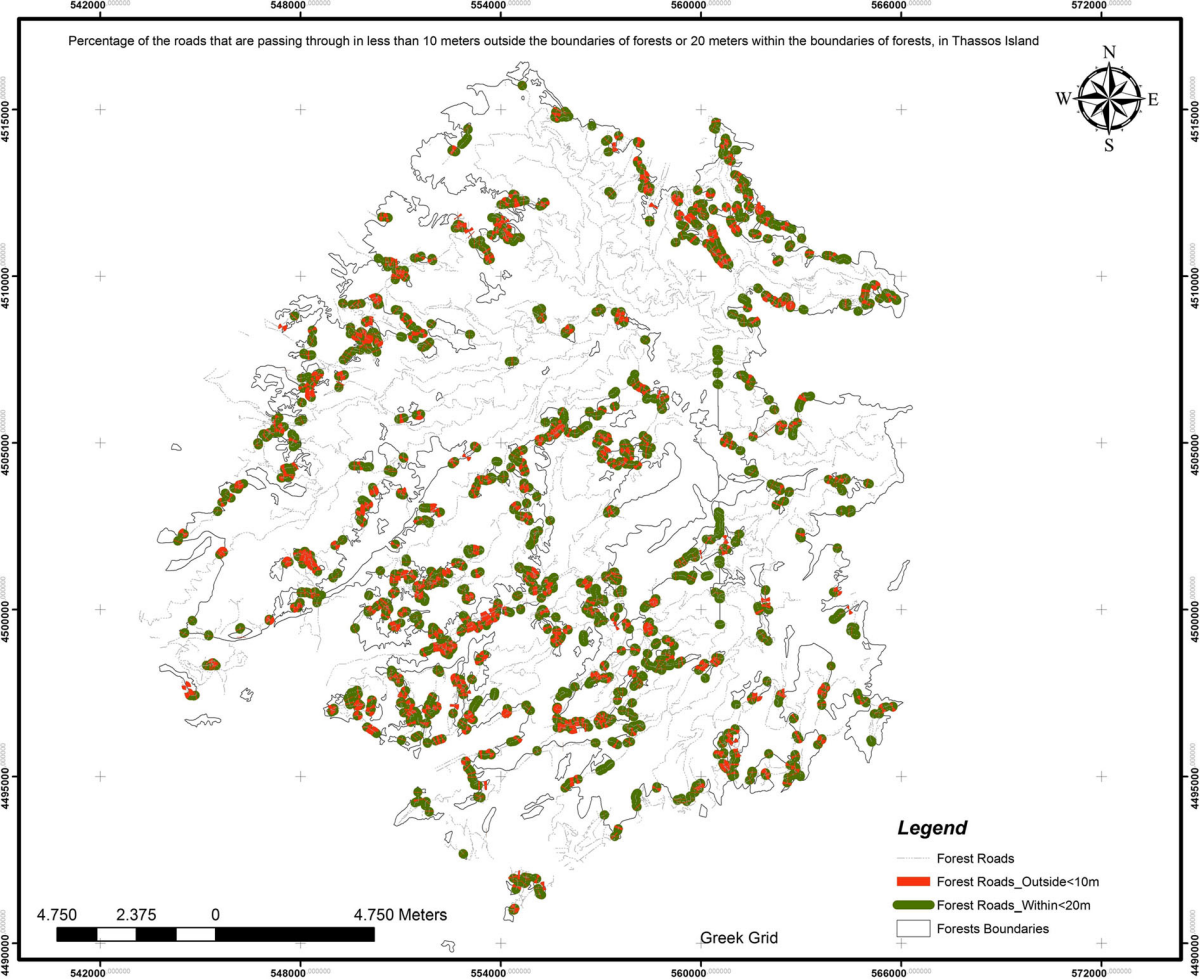
Percentage of the roads that are passing through in less than 10 m outside the boundaries of forests or 20 m within the boundaries of forests in Thassos Island

- The percentage of the roads that are passing through in less than 10 m outside the boundaries of forests or 20 m within the boundaries of forests (Fig. 6) is evaluated with the use of ModelBuilder tool of ArcGIS. Finally, the percentage of these roads is given in Table 2.

**Table 2:** The forest roads’ distance criterion evaluation from the forest’s boundaries (less than 10 m outside and 20 m inside)

Forest road distances from the forest boundaries	Forest roads’ length (m)	Road percentage (%)	Criterion evaluation (difference from optimum 100)	Criterion rate (average)
Forest roads inside boundaries in distance <20 m	50,797.8055	5.0598	94.9402	
Forest roads outside boundaries in distance <10 m	29,213.7161	2.9099	97.0901	
Rest of forest roads	923,940.8770	–	–	
Total	1,003,952.3985			96.0152

The criterion rate for the distance from the forest’s boundaries is 96.0152. Weighting factor, 3

**Fig 7:**
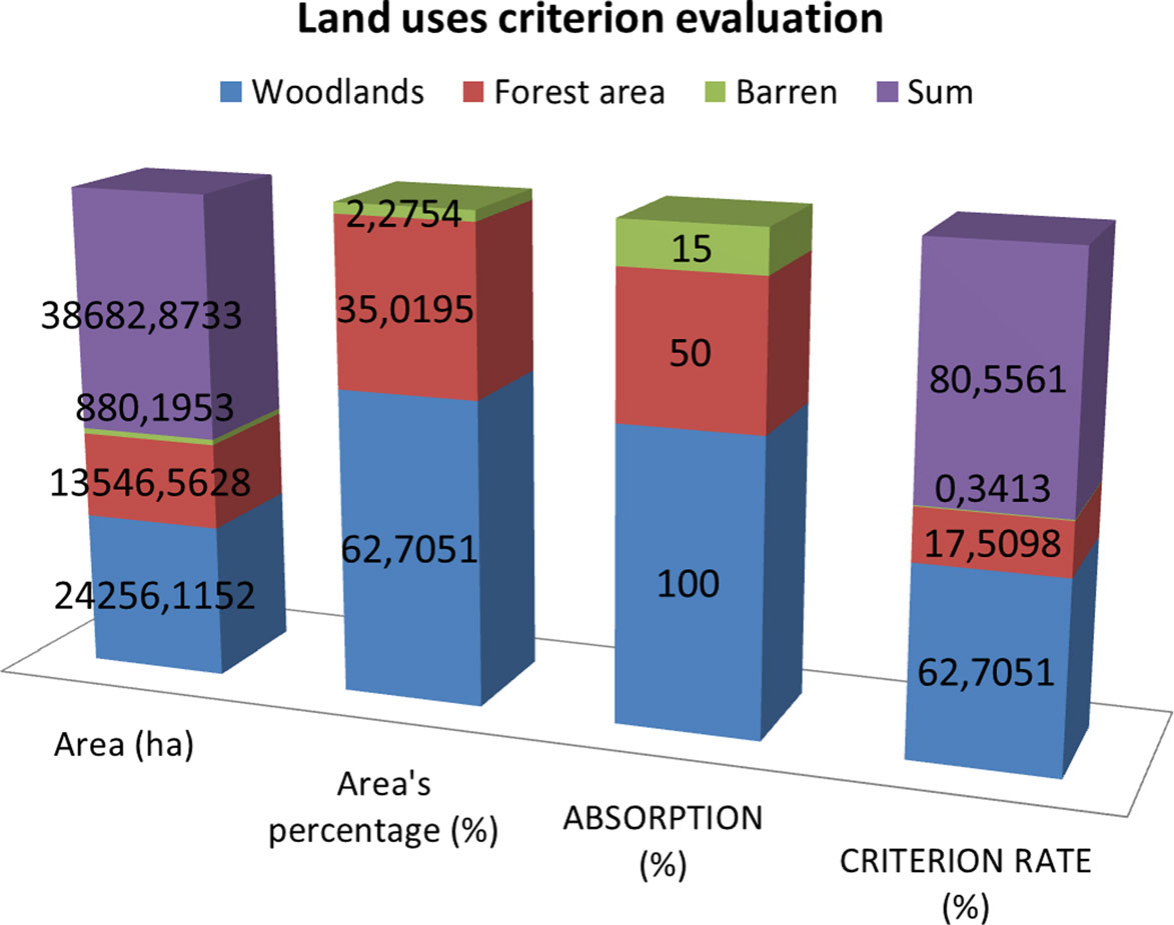
Land uses criterion evaluation

- The fieldwork results showed that the forest roads of Thassos island, as shown on the geological map (Fig. 7), do not pass through clay soils, large exposure streams, and unstable soils. So, the layout design rate of forest roads that pass through hazardous sites is rated 0 %. Thus, the criterion rate is 100 − 0 = 100 %. Weighting factor, 3

In Table 3, the average of the multicriteria intensity evaluation due the forest roads’ construction at the island of Thassos is presented.

**Table 3:** Multicriteria evaluation of intensity due the forest roads’ construction

Intensity			
Criteria	Rate	Weighting factor	Total
Road density and forest protection percentage	22.965	3	68.90
Applied skidding means	–	–	–
Skidding direction	–	–	–
Traffic load and truck type			
Excess percentage of the visitors’ number	100.000	2	200.00
Excess percentage due to overloaded vehicle wheels	100.000	2	200.00
Forest road location			
Forest road distance from the streams	96.941	3	290.82
Forest road distance from the forest boundaries	96.015	3	288.05
Forest roads that pass through hazardous sites	100.000	3	300.00
Total		16	1347.77
Average ΣI = Σ(I × *W*_I_) / Σ*W*_I_	1347.77/16 = 84.24 %		

### The rates of the absorption criteria

The rates of the absorption ability of the skidding consequences from the forest ecosystem are as follows:

#### Forestry criteria

**Fig. 8:**
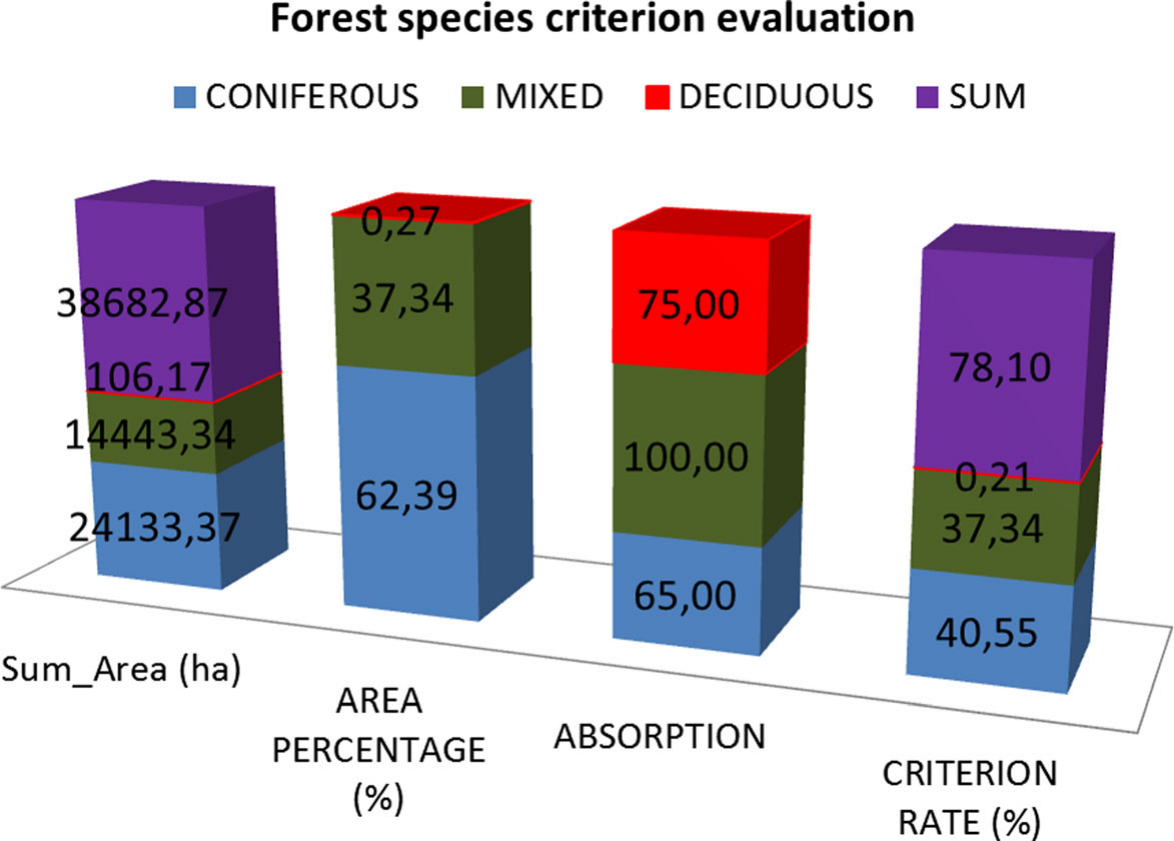
Forest species criterion evaluation
- The study area is covered by mixed trees 37.34 %, by broad-leaved trees 0.27 %, and coniferous trees 62.39 % (Fig. 2). Therefore, the absorption is 37.34 ∗ 1 + 0.27 ∗ 0.75 + 62.39 ∗ 0.65 = 78.096 % (Fig. 9).

- From the digitized map of land uses (Fig. 2), it is clear that the 62.7051 % of the study area is covered by forest and the 35.0195 % from forest area, and the 2.2754 % has no vegetation (barren). Therefore, the absorption is 62.7051 ∗ 1 + 35.0195 ∗ 0.5 + 2.2754 ∗ 0.15 = 80.5561 % (Fig. 8).

**Fig. 9:**
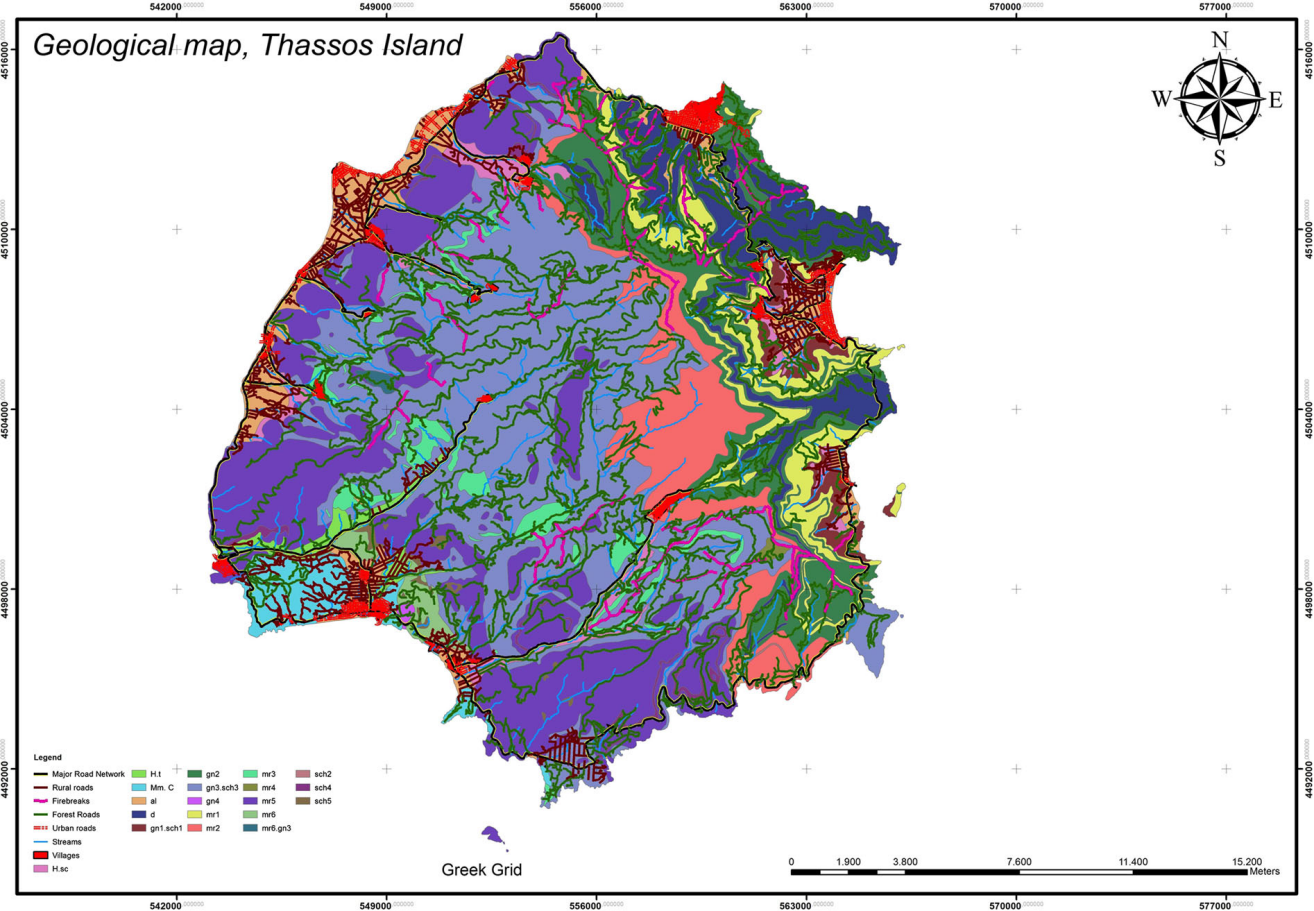
Geological map, Thassos island
- The forest management form is high forest (coniferous) 62.3877 %, coppice forest (mixed) 37.3378, and coppice forest (broad-leaved) 0.2745 %. Therefore, the absorption is 62.3877 ∗ 1 + 37.3378 ∗ 0.5 + 0.2745 ∗ 0.5 = 81.1939 %.
- The forest age is group-selective forest (coniferous) 62.39 %, even aged forest (mixed) 37.34, and even aged forest (broad-leaved) 0.27 %. Therefore, the absorption is 62.3877 ∗ 1 + 37.3378 ∗ 0.5 + 0.2745 ∗ 0.5 = 81.1939 %.
- The mean tree height arises for the 13 % from >20 m, for the 47 % from <10–20 m, and for the 40 % from <10 m; therefore, the absorption for this criterion is 13 ∗ 1 + 47 ∗ 0.75 + 40 ∗ 0.25 = 58.25 %.
- The plant index groups are Ι–ΙΙ, − (there is no soil of these classifications); III–IV, 1.1209 %; and V–VI, − (there is no soil of these classifications); therefore, the absorption is 1.1209 ∗ 0.5 = 0.560455 %.
- The forest productivity (annual growth) is smaller than 1 m^3^/year × ha, according to the last forest management plan which is 1–3 m^3^/year × ha. Therefore, the absorption is 100 ∗ 0.25 = 25 %.

#### Topographical criteria

For the extraction of the absorption values, we created slope and direction maps.

**Fig. 10:**
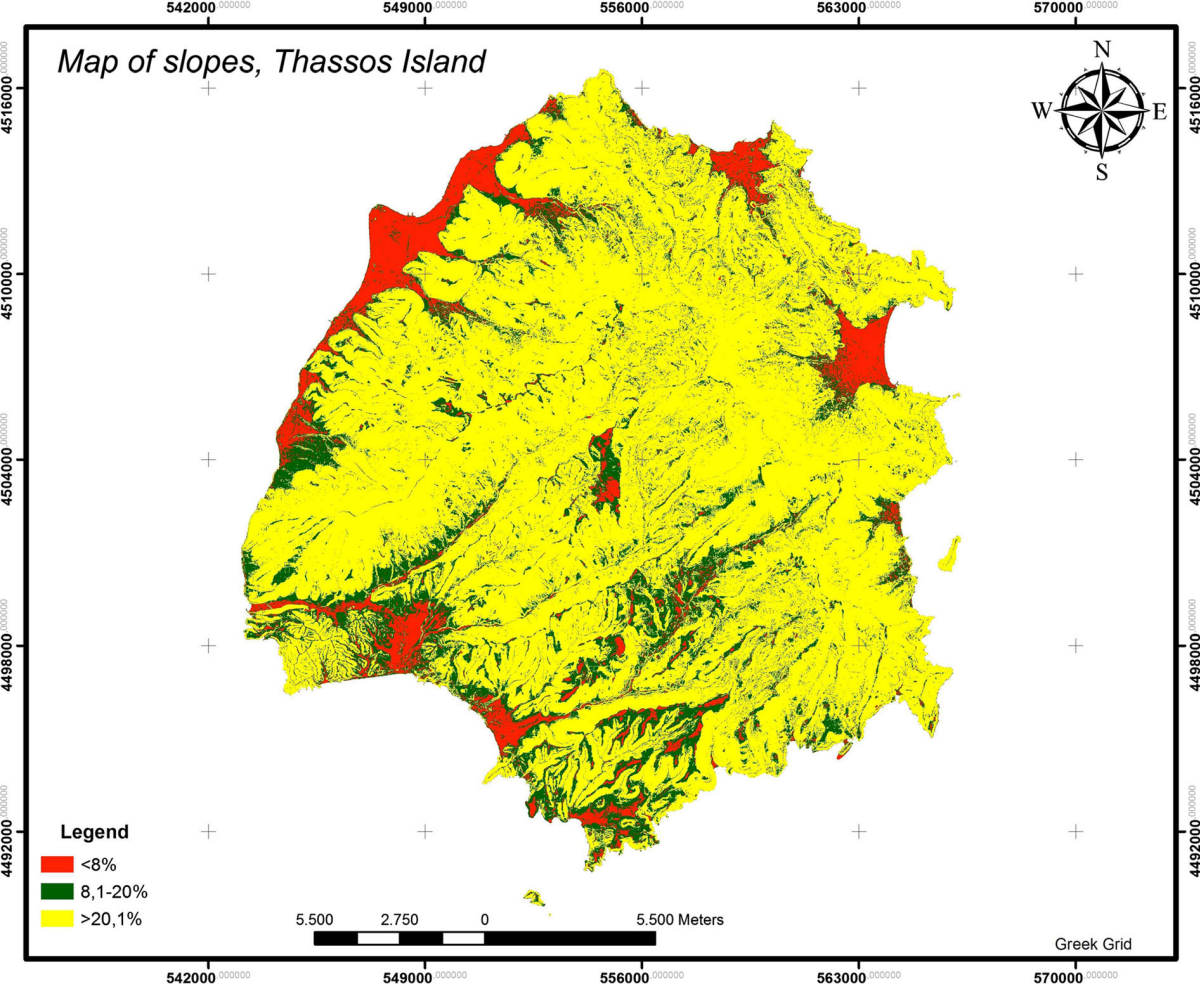
Map of slopes, Thassos Island
- The criterion evaluation that is related to the aspects for both elevation categories (<1000 and ≥1000 m) has been carried out with the use of the tool ModelBuilder (Fig. 11).

- The slope categories <8, 8–20, and >20 % correspond to the percentages 8.1838, 15.5070, and 76.3092 % respectively (Fig. 10). Therefore, the criterion value is 8.1838 ∗ 1, 15.5070 ∗ 0.5, and 76.3092 ∗ 0.01 = 23.56822 %.

**Fig. 11:**
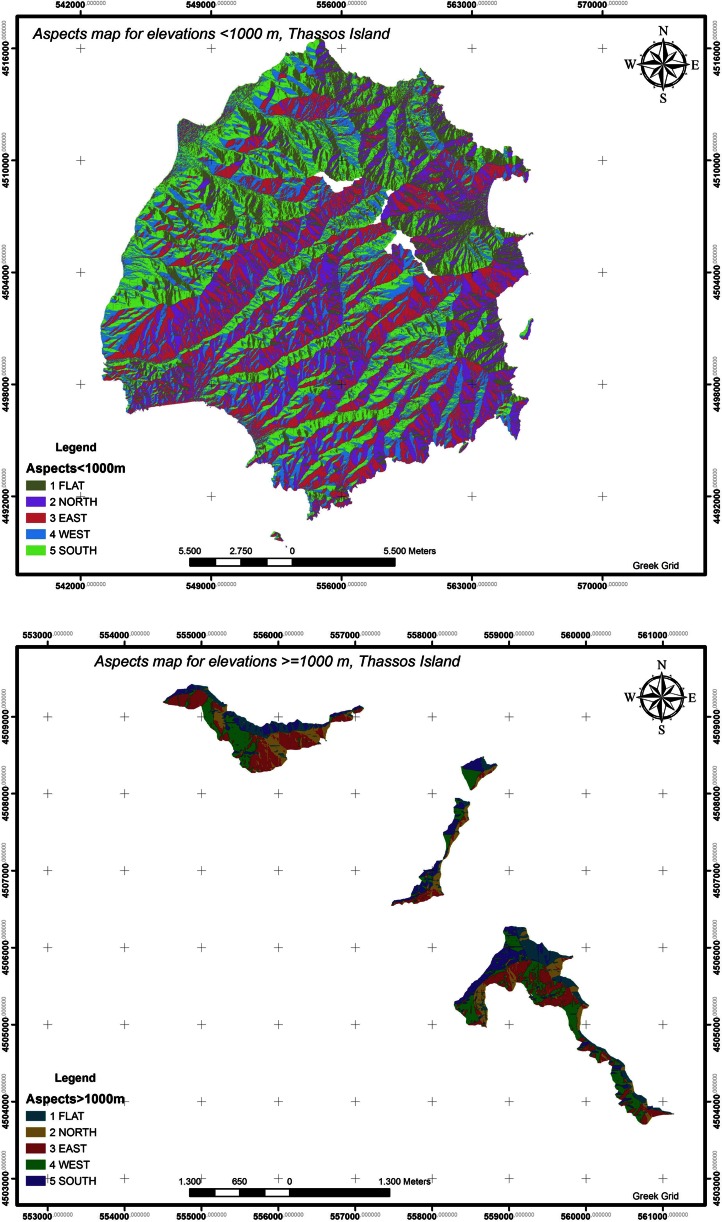
Aspects for elevation categories (<1000 and ≥1000 m), Thassos island
- For elevations <1000 m, the classification of aspects is as follows: FLAT 16.4443 %, NORTH 19.5061 %, EAST 21.9160 %, WEST 19.6986 %, and SOUTH 22.4350 %. Therefore, the criterion evaluation that is related to the aspects for elevations <1000 m is rated 19.5061 ∗ 1 + 21.9160 ∗ 0.75 + 19.6986 ∗ 0.75 + 22.4350 ∗ 0.50 = 61.9345 %.
- For elevation ≥1000 m, the classification of aspects is as follows: FLAT 14.9022 %, NORTH 15.0792 %, EAST 27.7994 %, WEST 26.05 %, and SOUTH 16.1691 %. Therefore, the criterion evaluation that is related to the aspects for elevations ≥1000 m is rated 15.0792 ∗ 0.7 + 27.7994 ∗ 1 + 26.05 ∗ 1 + 16.1691 ∗ 0.7 = 75.7233 %.
- Hence, for the final criterion assessment, the average has been calculated. Average criterion assessment = (75.7233 + 61.9345) / 2 = 68.8289 %.
- The terrain relief is described as intense with absorption value 15 %.

#### Social criteria

- The study area is a resort, since the whole island attracts many tourists each summer. Therefore, the criterion is rated 25 %. Weighting factor, 1
- In the study area, the existing national road network connects various settlements and villages and surrounds the island (Fig. 2). Therefore, the criterion is rated 25 %. Weighting factor, 1
- There is no railway network passing through the study area; thus, the criterion is not valued.
- In the study area, there is an archaeological area sited at the village Limenaria which is the capital of the island. Therefore, the criterion is rated 40 %. Weighting factor, 1
- On the island of Thassos, there is not any city as shown on Fig. 2; thus, the criterion is not valued.
- In the study area, there are some neighboring villages. Therefore, the criterion is rated 40 %. Weighting factor, 1
- There is no a European pathway in the study area; hence, the criterion is not valued.
- In the study area, there is the natural lake of Maries and some streams as shown in Fig. 2. Therefore, the criterion is rated 40 %. Weighting factor, 1

In Table 4, the average of the multicriteria absorption evaluation due the forest roads’ construction at the island of Thassos is presented.

**Table 4:** Multicriteria evaluation of absorption due the forest roads’ construction

Absorption			
Criteria	Rate (%)	Weighting factor	Total
Forestry criteria			
Land uses	80.556	3	241.668
Forest species	78.096	3	234.287
Forest management form	81.194	3	243.582
Forest age	81.194	3	243.582
Tree height	58.250	3	174.750
Soil quality	0.560	3	1.681
Forest productivity (harvesting)	25.000	3	75.000
Topographical criteria			
Slopes	23.568	2	47.136
Aspects	68.829	2	137.658
Terrain relief	15.000	2	30.000
Social criteria			
Tourist resort	25.000	1	25.000
National road network	25.000	1	25.000
Railway network	–	–	–
Archaeological area	40.000	1	40.000
Neighboring city	–	–	–
Neighboring village	30.000	1	30.000
European pathway	–	–	–
Natural or artificial lake or river	45.000	1	45.000
Total		32	1594.344
Average ΣA = Σ(A × *W*_A_) / Σ*W*_A_		1594.344/32 = 49.82 %	

By applying this method, the average of the environmental impacts’ intensity evaluation due the forest roads’ construction at the island of Thassos is ΣI = 84.24 %. Likewise, the average of the environmental impact absorption evaluation due the forest roads’ construction is ΣΑ = 49.82 % which is <50 %. Hence, by the assessment of the intensity and absorption criteria rates, we deduce that forest roads have not been constructed with the recommended method of the optimal spatial layout to the forest ecosystem. Also, the environmental impacts have not been absorbed by the natural environment. Additionally, the forest roads had not been constructed legally or according to the guidelines.

## Conclusions

This method plays a crucial role in the optimum solution selection (spatial, financial, forest, topographical, social, and environmental) for plan of forest road network. Additionally, this method constitutes the basis for a new decision support system (DSS) for the forest managers. It can also be customized to each area’s particularities and be applied for the creation of a new integrated decision support system (DSS).

The development and the application of the mapping of the spatial layout for the optimum forest roads’ network as well as the environmental impacts evaluation that are caused to the natural environment based on the MCE technique, with the use of the intensity and the absorption criteria evaluation, are an innovative tool.

The above conclusions are based on values that constitute indexes of environmental consequences from the forest roads’ planning and construction, to the natural environment. The application of this recommended method is considered to be reliable not only for the evaluation of the existing forest roads but also for the study of their impacts to the environment before the construction of new ones.

The usage of the GIS technology contributes to the application of the method for the evaluation of the intensity and absorption criteria. A suitable database is required for the application of the method. Thus, the data processing is achieved quickly and the creation of thematic maps and diagrams for various suggested road networks is evitable.

The application of an integrated development of the Mediterranean forest areas must be based on the sustainable development which depends on the preservation of the natural environment, the activation of the human and social resources, and the utilization of the special social, cultural, and financial characteristics that the Mediterranean ecosystems offer. For the achievement of the above development model, the recommended method plays a major role for mapping the spatial layout for the optimal forest road network and the environmental impacts evaluation that are caused to the natural environment based on the MCE technique.
